# Editorial: Racial health disparity in cancer: assessments of need

**DOI:** 10.3389/fonc.2023.1226697

**Published:** 2023-06-21

**Authors:** Jennie L. Williams, Jennifer A. Freedman, Camille Ragin, Folakemi T. Odedina, Patricia Thompson

**Affiliations:** ^1^ Stony Brook University, Stony Brook, NY, United States; ^2^ Stony Brook Medicine, Stony Brook, NY, United States; ^3^ Department of Medicine, Division of Medical Oncology, School of Medicine, Duke University, Durham, NC, United States; ^4^ Duke Cancer Institute, Durham, NC, United States; ^5^ Cancer Prevention and Control Program, Fox Chase Cancer Center, Philadelphia, PA, United States; ^6^ Mayo Clinic Comprehensive Cancer Center, Jacksonville, FL, United States; ^7^ Cedars Sinai Medical Center, Los Angeles, CA, United States

**Keywords:** cancer disparities, determinants of cancer burdens, cancer health equity, black indigenous and people of color (BIPOC), structural and systemic racism

Globally, societal inequities and inequalities impact human health and contribute directly to the disparities in cancer incidence, progression, and outcomes between populations. In the United States (US), this includes inequities and inequalities that permeate all aspects of cancer care and control. Disparities are observed across the entirety of the cancer care continuum, including etiology, prevention, early detection, diagnosis, interception, treatment, survivorship, and end-of-life care. This results in higher cancer-related morbidity and mortality in Black, Indigenous, and people of color (BIPOC), people living in poverty and/or rural communities, persons with disability, and individuals who are part of LGBTQA+ communities. This problem persists nearly a century after the initiation of legislation establishing NCI (1937) and despite extensive advances in surveillance mechanisms, prevention, early detection, diagnosis, and treatment of many cancers. With the establishment of SEER in 1973, gender, racial and ethnic disparity have been detected and continue to be monitored. However, it took 27 years after the establishment of SEER before a national concerted effort was put forth to address and mitigate cancer disparity under the purview of NCI Center to Reduce Cancer Health Disparities. And while cancer is declining with advocacy, community partnership, and investment in research and education, African American/Black patients in the US continue to have the highest cancer mortality rates and shortest survival for most cancers (1). In contrast, cancer incidence rates have historically been lower for the US Hispanic/Latino and Asian populations. However, disparities including younger age onset of certain cancers [e.g., colorectal among Hispanics/Latinos (2), breast cancer among Asian women (3)], more infection-related cancers among US Hispanics/Latinos (4) and Asian Americans (5), and disproportionate late-stage diagnoses for some cancers and among subpopulations (4, 5) are emergent concerns; which is further acerbated by the projected growth of these populations.

While racial- and ethnicity-related cancer disparities are often framed in terms of differences in access to care and individual- or group-level adherence to health behaviors, this can be highly counterproductive in efforts to understand, address, and mitigate cancer disparities. To effectively address this concern, it is important to recognize that individual-level, ancestry-related (e.g., genetics, genomics, and physiology), societal-level (e.g., structural and systemic racism), social-level (e.g., socioeconomic status and educational level), neighborhood-level (e.g., diet and pollution), and institutional-level (e.g., access to care) factors act together and intersect to influence not only an individual’s health but also the health of the community, and, therefore, collectively contribute to health disparities that arise therein. With the rapid diversification of the racial and ethnic composition of the US population, including growth among US Hispanics/Latinos, Asian Americans, and individuals of mixed races/ethnicities, wider adoption of methods to disaggregate race and ethnicity to account for individual-level, ancestry-related, societal-, social-, neighborhood-, and institutional-level factors that differ within and between groups will be needed to understand, address, and mitigate the burden of cancer more precisely in communities. These methods will need to include partnering with diverse groups in basic, translational, and clinical cancer research and education. BIPOC, LGBTQA+ populations, rural communities, and low-income people remain underrepresented in cancer research, including genomic projects and clinical trials.

While it is acknowledged that an in-depth ‘omics’ understanding of the molecular differences in cancers that develop in different populations and communities and how these differences can inform cancer etiology, prevention, detection, diagnosis, interception, treatment, survivorship and end-of-life is essential for advancing and achieving cancer health equity, it is also necessary to understand all of the individual-level, ancestry-related, societal-, social-, neighborhood-, and institutional-level factors that act solely and in concert throughout the natural history of cancer in communities and how these contribute to cancer health disparities. This Research Topic was intended to solicit unpublished research on cancer disparities in racially and ethnically diverse populations. We were looking to generate a publication highlighting the multiple factors that solely and in combination influence cancer and cancer burden at the community-level and contribute to differences between population groups. This included focusing on individual-, ancestry-related-, societal-, social-, neighborhood-, and institutional-level determinants of cancer burdens in different populations and how they individually and jointly contribute to cancer disparities. This Research Topic aimed to promote and advocate for more research efforts toward understanding and addressing the many drivers of cancer health disparities toward ultimately aiding in mitigating cancer health disparities. Nine reports of original cancer research, a perspective, a mini-review, and a methods paper are all included in this Research Topic. Together, these efforts draw attention to the many often interrelated factors (e.g., genetic, genomic, screening, environment, socioeconomic, and racism) contributing to cancer disparities. The works of these authors and their teams also emphasize the greater need to engage racially, ethnically, culturally, and sexual/gender diverse individuals and communities in cancer-relevant research and education as an essential step towards understanding and addressing the cancer burden in communities to advance evidence-based strategies to achieve cancer health equity for all individuals, their families, and their communities.

## Author contributions

All authors listed have made a substantial, direct, and intellectual contribution to the work and approved it for publication.
